# Carfilzomib and dexamethasone for extramedullary myeloma with pleuropericardial involvement

**DOI:** 10.1002/ccr3.1015

**Published:** 2017-06-22

**Authors:** Ignacio Español, Marta Romera, María Dolores Gutiérrez‐Meca, Maria del Carmen García, Aurelia Tejedor, Antonio Martínez, Jerónima Ibáñez, Felipe De Arriba, Alfredo Minguela, Teodoro Iturbe, Maria Dolores López

**Affiliations:** ^1^ Haematology Department Santa Lucía University General Hospital Cartagena Spain; ^2^ Hematology Department Morales Meseguer University General Hospital Murcia Spain; ^3^ Immunology Department Virgen de la Arrixaca University General Hospital Murcia Spain

**Keywords:** Cardiac tamponade, carfilzomib, pleuropericardial myelomatous effusion

## Abstract

The dismal outcome of pleuropericardial extramedullary multiple myeloma (EMM) reflects both the selection of resistant disease and absence of useful drugs. Carfilzomib and dexamethasone should be explored in advanced EMM patients, even for bortezomib‐resistant patients, as may provide much longer overall survival than previously reported treatments.

## Introduction

Multiple myeloma is a malignant mature B‐cell neoplasm characterized by multifocal plasma cell proliferation associated with a serum and/or urine M‐protein. Although symptomatic multiple myeloma is usually restricted to the bone marrow with ≥ 10% clonal plasma cell infiltration, it can present as an extramedullary disease, mainly plasmacytomas. Extramedullary multiple myeloma (EMM) is an unusual event occurring at diagnosis or, more frequently, during disease progression or relapse 1. Pericardial or pleural myelomatous effusions as EMM involvements are extremely rare, occurring in less than 1% of cases, and usually correspond to advanced stages of the disease. So far, there are no efficient treatment options for these patients. Therefore, the prognosis is dismal with < 3 months of average survival 2.

Here, we report the case of an EMM with pericardial effusion, cardiac tamponade, and pleural myelomatous infiltration. The patient responded successfully to pericardiocentesis, thoracocentesis and a treatment with carfilzomib and dexamethasone achieving a durable complete remission.

## Case Report

A 39‐year‐old woman was diagnosed with plasmablastic IgA *lambda* multiple myeloma, stage III (International Staging System) in January 2011. Bone marrow examination showed a huge infiltration (80%) of immature plasma cells and a fluorescence in situ hybridization (FISH) assay revealed t (4;14) (29%), del (13) (q14) (27%), and disruption of *IgH* (14q32) (27%). The patient was treated with four cycles of PAD chemotherapy (bortezomib, adriamycin, and dexamethasone) and zoledronic acid achieving a stringent complete remission. Then, an autologous peripheral blood stem cell transplant, conditioned with melphalan (200 mg/m^2^), was performed in August 2011.

As a symptomatic relapse with a T7 vertebral collapse was detected in March 2013, treatment with lenalidomide and dexamethasone was started. However, after four cycles of treatment a loss of response was observed. Therefore, the patient received VCD chemotherapy (bortezomib, cyclophosphamide, and dexamethasone), achieving a second complete remission. A fully matched sibling hematopoietic stem cell transplantation, after reduced‐intensity conditioning regimen, was performed in December 2013, trying to consolidate the excellent response. Post‐transplant chimerism studies showed a donor immune restitution.

Another bone marrow relapse, along with a plasmacytoma of tibia, was diagnosed in April 2014. Immunosuppressive therapy was discontinued, and the patient was retreated with VCD. This regimen was stopped after two cycles of treatment due to bortezomib‐related peripheral neuropathy. The patient was then placed on VBCMP/VBAD chemotherapy (vincristine, carmustine, cyclophosphamide, melphalan, prednisone/vincristine, carmustine, doxorubicin, and high‐dose dexamethasone), achieving a third complete remission.

**Figure 1:**
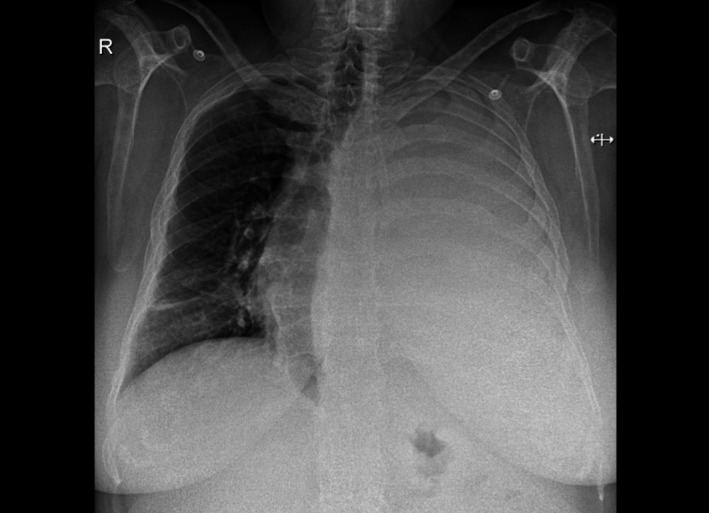
Massive left‐side pleural effusion on chest X‐ray.

**Figure 2:**
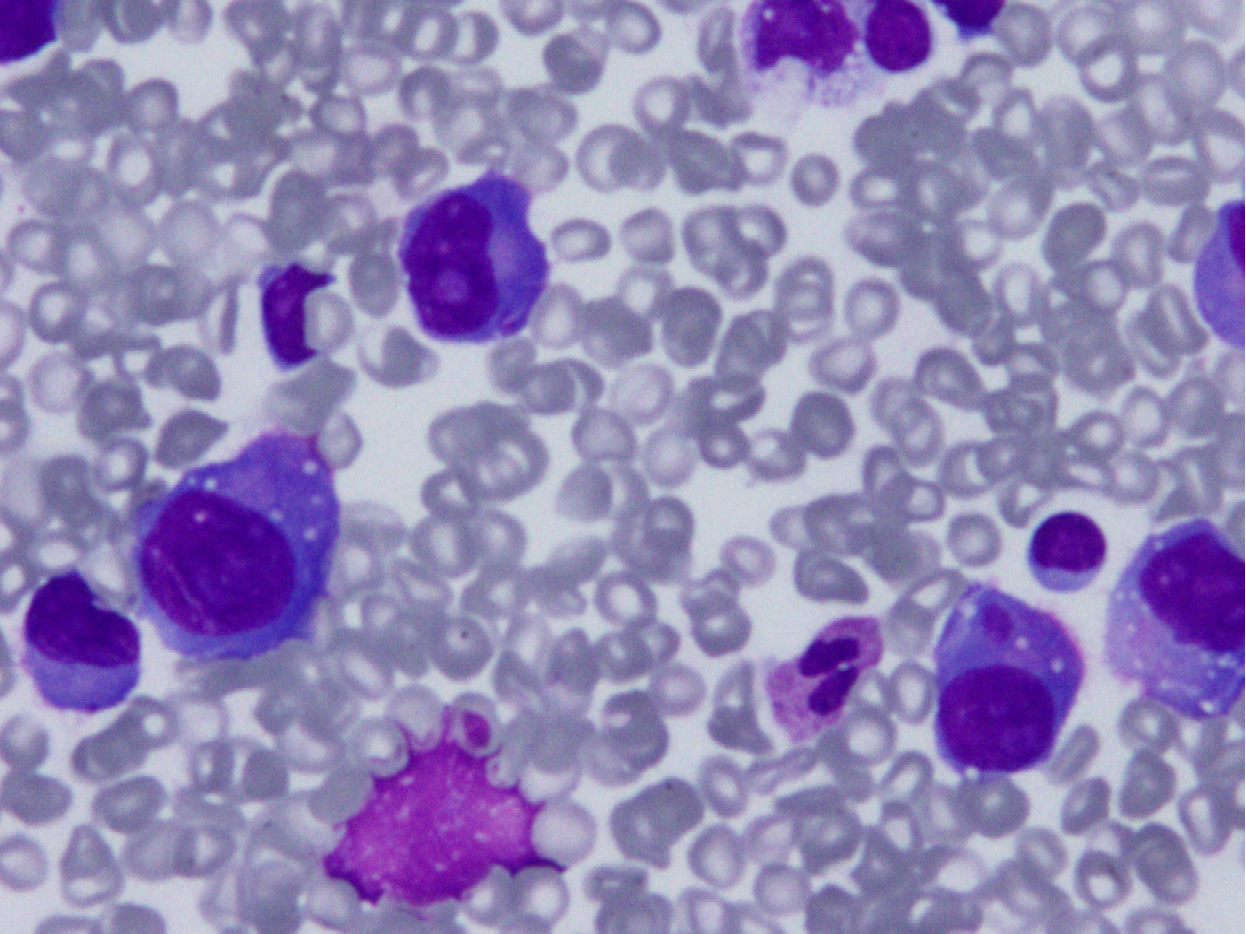
Hemorrhagic pleural fluid with cells resembling plasmablasts with eccentrically placed nuclei, prominent nucleoli, and large basophilic cytoplasm. May‐Grünwald‐Giemsa staining.

In April 2015, she presented shortness of breath, chest pain, and pericardial effusion with echocardiographic signs of cardiac tamponade. Pericardiocentesis drained 300 mL of hemorrhagic fluid with cells that seemed plasmablastic cells. No further pericardial diagnostic studies could be performed. However, no plasma cells were detected in bone marrow or peripheral blood and there was no evidence of serum or urine paraprotein. Free light chain ratio was normal. Amyloidosis was also ruled out by rectal biopsy. The patient was discharged without symptoms. One month later, she presented with breathlessness and tachycardia and a chest X‐ray showed massive left‐side pleural effusion (Figure 1). Two thoracocentesis drained 2.5 L of heavily blood‐stained fluid, revealing plasmablastic cells (Figure 2) that were confirmed by flow cytometry immunophenotypic analysis. Despite this EMM relapse, multiple myeloma was not detected in blood, urine, or bone marrow.

The patient started a sixth line of treatment with carfilzomib plus dexamethasone. Carfilzomib, 20 mg/m^2^, was administered intravenously over 10 min on days 1, 2, 8, 9, 15, and 16 of each 28‐day cycle. Oral dexamethasone, 4 mg, was given before each dose of carfilzomib. The patient experienced a sudden improvement in her general condition and was discharged without pleural effusion. Carfilzomib dose was escalated to 27 mg/m^2^ for all cycles thereafter. Treatment tolerance was excellent. A new echocardiography showed no pericardial effusion. The patient is currently receiving the 18th cycle of treatment and remains in complete remission 19 months after the EMM was detected.

## Discussion

Symptomatic multiple myeloma is mostly restricted to the bone marrow, presenting anemia, hypercalcemia, or painful bone lesions. However, multiple myeloma plasma cells may be detected in extramedullary sites, such as liver, skin, or brain. The incidence of EMM ranges from 2% to 20% and is mostly observed upon disease relapse or progression 1. EMM may be bone related, when remains connected to the bone, or soft tissue related. The latter has a shorter overall survival. Pericardial or pleural myelomatous effusions as extramedullary disease involvements are extremely rare 2, 3. Abelman et al. 2 reviewed pericardial myelomatous disease revealing a median age at presentation of 67 years, with IgG isotype predominance; 67% of cases were relapsed myeloma patients, cardiac tamponade was present in 63%, and pleural involvement was seen in 19% of these patients. Prognosis was poor with progression or disease recurrence after 3 months in 65% patients. Moreover, Cho et al. reviewed pleural myelomatous effusions describing a median age at diagnosis of 58 years, with higher frequency of IgA and IgD isotypes. Most cases also presented bone marrow infiltration. The prognosis was also poor, with median survival of 2.8 months 3.

Here, we present a relapsed IgA *lambda* multiple myeloma patient who underwent five lines of chemotherapy and two hematopoietic stem cell transplants. She developed a myelomatous pericardial effusion with cardiac tamponade followed by a severe myelomatous pleural effusion. The extramedullary involvement was nonsecretory and was not accompanied by bone marrow infiltration. In the literature, pericardial infiltration has been reported in patients with myelomatous pleural infiltration 3 and in multiple myeloma patients treated with high‐dose therapy such as stem cell transplantation 4. The mechanisms underlying the spread of plasma cells outside the bone marrow are poorly understood and may involve bone marrow hypoxia and altered expression of chemokines and adhesion molecules that regulate plasma cell adhesion to bone marrow microenvironment 5.

Identification of plasma cells must be performed by microscopic examination and flow cytometry studies of the pleuropericardial fluid. The presence of plasmablastic cells in pleuropericardial fluid should alert clinicians to the diagnosis and the need to initiate quick and aggressive therapy with new drugs. EMM at relapse and/or associated with soft tissues is more aggressive than when present at diagnosis and/or bone related. Our patient fulfilled several adverse prognosis characteristics, as the EMM was detected in a late relapse, affecting only soft tissues, that is, pericardium and pleura, and displaying chromosomal alterations related with the development of EMM, such as t (4;14), del (13)(q14), and disruption of *IgH* (14q32) 6.

Proteasome inhibitors, immunomodulatory drugs, and monoclonal antibodies in multiple myeloma have improved response and overall survival rates. Despite such advances, most patients ultimately relapse. Carfilzomib is a second‐generation proteasome inhibitor that has demonstrated efficacy, safety, and tolerability in patients with relapsed and/or refractory multiple myeloma 7. EMM patients are treated with systemic chemotherapy, mainly VAD, CHOP, or melphalan and prednisone schemes 5. Intrapericardial steroids and/or chemotherapy have also been tested, as well as radiotherapy 4. However, patients with pleuropericardial effusions are usually resistant to treatment and often relapse in spite of aggressive chemotherapy 8. Median time to death from diagnosis of pericardial involvement is 13 weeks and patients die mainly from progressive disease (92%) and/or related sepsis 2. Our patient was treated with pericardiocentesis, thoracocentesis, and a combination of carfilzomib and dexamethasone achieving a quick and sustained complete response, with excellent tolerance. In carfilzomib‐naïve patients, this regimen may be effective, even for bortezomib‐resistant patients.

In summary, this is the first reported case of a long‐lasting complete response of an EMM with pleuropericardial myelomatous infiltration, an otherwise lethal complication. Treatment with carfilzomib and dexamethasone showed a rapid and durable response, without side effects. Therefore, carfilzomib plus dexamethasone should be explored in advanced, bortezomib‐resistant EMM patients, as may provide much longer overall survival than previously reported treatments.

## Authorship

IE: wrote the manuscript. MR, MDGM, MCG, AT, AM, JI, TI, MDL: took care of the patient. AM: performed the immunophenotypic studies. FDA: performed the stem cell transplantation.

## Conflict of Interest

The authors declare that they have no conflict of interest.
